# Green bonds issuance: insights in low- and middle-income countries

**DOI:** 10.1186/s40991-020-00056-0

**Published:** 2021-01-04

**Authors:** Ursule Yvanna Otek Ntsama, Chen Yan, Alireza Nasiri, Abdel Hamid Mbouombouo Mboungam

**Affiliations:** 1 School of Accounting, Dongbei University of Finance and Economics, No. 217 JianShan St., Shahekou District, Dalian, 116025 China; 2 School of Accounting, Dongbei University of Finance and Economics, No. 217 JianShan St., Shahekou District, Dalian, 116025 China; 3 University of Tehran, Tehran, Iran; 4 Northwestern Polytechnical University, 1 Dongxiang Road, Chang’an District, Xi’an, 710129 Shaanxi China

**Keywords:** LMIC’s, Green bonds, ESG, Sustainability investment

## Abstract

Former reports of Environmental, Social and Governance (ESG) tended to focus on the equity side of investing, and today green bonds also offer and introduce sustainability factors. This paper is about the relevance, potential benefits and key arguments for countries with low-and middle-incomes where financial markets are not comparable with those in developed countries. We begin by stating clearly the relevance of a green economy transformation, highlight the development challenges they face and talk about how a green economy approach can help to solve these challenges. Then an outline of the progress that has been made in this panel, and the economic and social benefits that a green economy can potentially offer to Low- and Middle-Income countries (LMIC’s) will be underlined. Finally, we will make recommendations on the range of potential areas for intervention.

## Introduction

The global warming has increased per decade since 1880 (National Centers for Environmental Information, 2019) and has brought many concerns throughout economists and market participants. The part of the globe that has warmed since 1880 represents 98%; this suggests new risks that affect the economy. The first to merge global warming into endogenous growth models was Nordhaus (1990); but it is with the Stern review, (Stern, 2006) drawn up at the request of the British government, that economic theory really enters the debates on climate policy. The investments required to mitigate these disruptions are massive. Knowing that public investment cannot be a solution on its own, private capital will be essential to finance the green economy and the innovations that must accompany it. In response to this need of liquidity, the famous Green Bonds (considered as viable alternative to traditional financing mechanisms) have been launched to finance projects that respect certain environmental criteria.

Around 1970, Socially Responsible Investing (SRI) was a curiosity and a niche market. It has since become a global movement and has entered the vocabulary and consciousness of the world of finance (Sparkes, 2002). The first green bond was issued in 2008 and was carried out in Europe. It is especially since the 2015 Paris Climate Agreement that the green bond market has grown remarkably, despite the lack of a precise definition of the object financed by these bonds that would be recognised by all players at the international level. Globally, green bond issues hit a record of more than $250 billion in 2019 (Climate Bonds Initiative, C., 2020). Despite the current crisis related to Covid-19, the trend for this product, as well as for the bonds of the same family such as social and sustainable obligations, remains that of an increasingly large and diverse growth. Green bonds are among the options available for private sector companies and public institutions committed to support climate and environmentally friendly investments. In 2019, the financial performance of the green bond market was supported by the overall downward movement in rates: the most followed index by managers, the Bloomberg Barclays MSCI Global Green Bond Index, gained 6.50% over the year, after a year 2018 in the red (− 0.72%). According to figures compiled by the Climate Bonds Initiative (Climate Bonds Initiative, C., 2020), green bond issues broke records in 2019: $255 billion (231 billion Euros) of new securities added to the market, up from $167 billion in 2018. And the trend is expected to continue in 2020, with $350 billion to $400 billion in emissions expected (Filkova & Almeida, 2019).

For the past 4 years, in the wake of the Paris Agreement, 2015, green bond markets expanded strongly. They differ from conventional bonds by the expected environmental benefit of the funded project. However, it falls short of the financing needs of the ecological transition. In addition, the absence of a precise legal definition of financing objects, create a risk of greenwashing for the issuer and increase the cost of information on the nature of the project for the investor.

The growth prospects of the Green Bonds market are encouraging with many government initiatives in low- and middle-income countries to support the development of this market. The need to standardize the eligibility criteria for Green Bonds is certainly one of the biggest challenges facing this still-recent market. Despite the multiple difficulties faced by low-and middle-income countries (poverty, political conflicts, and ecosystem degradation), with Africa being the most vulnerable one to climate-change policies, implementation of green capital continues to make its way. Africa’s green bond issuance is 0.18% of its total market capitalization, compared to 0.4% in North America (US& Canada), 1.9% in Eurozone, 0.89% in China (World Bank Group 2019). Therefore, the successful development of green bond markets entails considering several different factors.

It should be noted here that the focus will not be on African countries because of the relatively small size of African financial markets compared to other regions. As the Green, Social and Sustainability Bonds market is a growing market, private investors are more likely to invest in global capital markets, where they have a better social, economic and political context control. At the global level despite the recent development of finance, countries such as France, Sweden, Switzerland, China, and some states of the United States of America, are leaders in the field with the establishment of a regulatory framework and also the issuance of several green and sustainable bonds. Climate finance in 2017/2018 has increased by 25% over previous years, thanks to the rapid growth of installed renewable energy capacity in China and India, the growing commitment to better land use and energy efficiency in many parts of the world. By region, East Asia and the Pacific remains the top destination for climate finance (this region received $238 billion a year, or 41% of the climate finance tracked. A trend mainly due to China which is the largest provider and the first destination of climate finance, for several years). The fastest growth in climate finance has been observed in Oceania, the Middle East and North Africa. The climate funding received by Oceania in 2017/2018 is equivalent to 165% of the amounts received in 2015/2016. There is also a 78% growth in North Africa over this period.

The paper is a summary of recent literature, aiming to generalize relevant academic researches and seeks to provide an introduction to green bonds for new scholars. Secondly, it attempts to give a platform for further green finance research by delineating the major financial, practical and political concerns with green bonds in low-and middle-income countries. Finally, it aims to enlarge our knowledge of the green bond market by putting critical research agendas into direct conversation. The paper concludes by calling for more explicit analysis of green bonds in low-and middle-income countries. By learning from the current status, we hope to form a proper understanding of the conceptual issues, identify the development trends and provide useful guidance on future research directions in this important and developing subject.

## Methodology

**Fig. 1:**
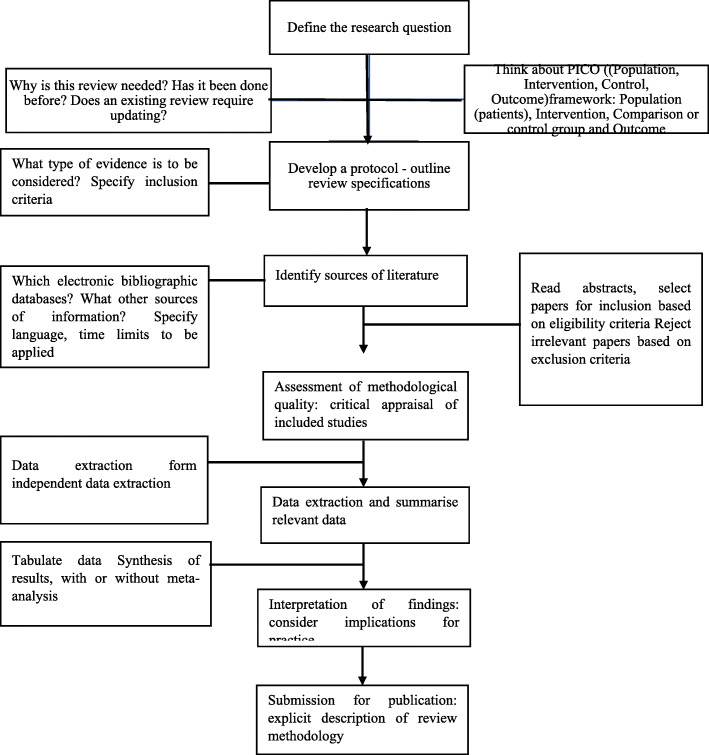
Research design for a systematic literature review

The method used in this paper is a systematic analysis approach. A systematic review is a critical synthesis of research evidence, which involves analysis of all available and relevant evidence in a systematic, objective and robust manner (Fig. 1).

As well as mathematics education, it can be applied to numerous disciplines (Schwarz, 2015) and can be used both to develop theories, describe social phenomena or test hypotheses (Hopf, 2016). For this purpose, a special focus should be paid on the formation of categories and codes that are important for an effective research (Kuckartz, 2019).

## Literature review

### Socially responsible investing

The research on SRI has increased over the last decade. From 1900 to 2017, a total of 634 SRI articles has been recorded (Widyawati, 2019). Socially Responsible Investment is a hybrid form of investing that covers important criteria (Environmental, Social or Governance) grouped under the acronym ESG (Avetisyan & Hockerts, 2017; Friede, 2019) aiming to achieve sustainable development goals (PRI, 2017). Responsible investing is done in two ways: the first is to eliminate companies whose balance sheets are not considered to be responsible (this approach is the main one in Europe). The second approach is to rate companies on ESG criteria and invest more in companies with the best ESG profile Without completely excluding less responsible companies (this approach is the main one in the USA). However, the concept of SRI is extremely complex and individualize from one investor to another (Sandberg et al., 2009; Juravle & Lewis, 2008).

### From sustainable finance to green finance

The term sustainability is “a capacity to support some entity, outcome, or process over time” (Jenkins, 2009) and carrying out activities that do not exhaust the resources on which that capacity depends.

Sustainable Finance refers to financial practices that take into account extra-financial reporting including social, environmental, societal and governance information on corporate activity. It includes Socially Responsible Investing (SRI), which has had its own label since August 2016, and has served to companies that met the ESG (Environmental, Social and Governance) criteria in a sustainable way. Indeed here, the concept of environment and sustainable development is present, but also social criteria (such as respect for gender parity) or governance criteria (such as transparency in executive pay).

Green finance covers all services offered in financial markets to invest in initiatives to reduce the impact of human activities on the environment or to offer social, economic and environmental benefits. The main tool of green finance remains green bonds (Table 1).

**Table 1:** Overview of reports on Green Finance

Authors/publication and year	Meaning and understanding of Green Finance
(Höhne, Khosla, Fekete, & Gilbert, 2012)	“Green finance is a broad term that can refer to financial investments flowing into sustainable development projects and initiatives, environmental products, policies that encourage the development of a more sustainable economy, and to a wider range of environmental objectives.”
(Zadek & Flynn, 2013)	“Green finance is often used interchangeably with green investment. However, in practice, green finance is a wider lens including more than investments as defined by Bloomberg New Energy Finance and others. Most important is that it includes operational costs of green investments not included under the definition of green investment. Most obviously, it would include costs such as project preparation and land acquisition costs, both of which are not just significant but can pose distinct financing challenges.”
(PricewaterhouseCoopers, 2013)	“For the banking sector, green finance is defined as financial products and services, under the consideration of environmental factors throughout the lending decision making, ex-post monitoring and risk management processes, provided to promote environmentally responsible investments and stimulate low-carbon technologies, projects, industries and businesses.”
(Hens et al., 2016)	“According to our definition, “Green Finance” comprises all forms of investment or lending that take into account environmental impact and enhance environmental sustainability. A key element of Green Finance is sustainable investment and banking, where investment and lending decisions are taken on the basis of environmental screening and risk assessment to meet environmental sustainability standards.”

### Overview of green bonds markets

#### Typology of green bonds

Bonds can be used to finance or refinance a variety of projects and activities, such as infrastructure, power plants or maintaining ongoing operations (Weber & Feltmate, 2016). A green bond is a debt security issued by a government entity, a multilateral institution, or a corporation to raise capital from investors for a project that contributes to a low-carbon, climate-resilient economy (Inderst et al., 2012). Green bonds are fixed-income instruments with proceeds earmarked exclusively for new and existing projects that have environmental benefits (Syzdykov & Masse, 2019). The term ‘green bonds’ refers to bonds whose proceeds are used to finance environmentally-friendly projects (Mercer, 2015) such as renewables, water and energy efficiency, bioenergy, and low carbon transports (Campiglio, 2016). They not only encompass financial obligations but also incorporate environmental benefits claimed by the green bond issuer (Bartels et al., 2016). Given the ESG shortfalls and other considerable factors, investors are offered a reasonable way to positively support sustainability while investing in a relatively low risk / low yield instrument (Kidney et al., 2012).

There are six types of green bonds (Table 2) referred as followed:

**Table 2:** Typology of Green Bonds

Green Bond Type	Attribute	Debt recourse
Use-of-Proceeds Bond	Proceeds raised by bond sale are earmarked for green projects in the issuer’s portfolio.	Recourse to the issuer. Entire balance sheet
Use-of-Proceeds Revenue Bond or ABS	Proceeds raised by bond sale are earmarked for or refinances green projects	Recourse is limited to an issuer’s pledged revenue streams.
Project Bond	Proceeds raised by bond sale are earmarked for a specific project.	Recourse is restrained to the project’s assets and balance sheet
Securitisation (ABS) Bond	Proceeds raised by bond sale are pooled are earmarked for green projects	Recourse is to a group of projects that have been grouped together
Covered Bond	Proceeds raised by bond sale are earmarked for eligible projects included in the covered pool	Recourse either to the issuing entity or to an affiliated group to which the issuing entity belongs and to a pool of collateral that is separate from the issuer’s other assets
Loan	Proceeds raised by bond sale are earmarked for eligible projects or secured on eligible assets	Recourse is full to the borrower in the case of unsecured loans. Recourse to the collateral in the case of secured loans

Source: (Climate Bonds Initiative, 2018), (Banga, 2019), (Berensmann et al., 2018)

#### Development of green bonds markets

The first green bond was issued in 2007 by the European Investment Bank (EIB), which was followed in 2008 by the International Bank for Reconstruction and Development (IBRD) (Coston et al., 2014; Stoian & Iorgulescu, 2019). The green bond market grew from $1.5 billion in 2007 to $389 billion in 2018 (CBI 2018c, 2018). Since its launch, the Green Bond has been a resounding success.

Far from their original containment to energy and water management, they are now linked to land use, waste management or sustainable transport. They also attract more exotic finances; the first Islamic Green Bond was issued in July 2017 in Malaysia. In 2015 some of the countries listed in this study joined the green bond market such as: Brazil, Denmark, Estonia, China, India, Latvia, and Mexico, contributing to a total annual issuance of $41.8 bn. According to Kreivi (2017), Director and Head of Capital Markets Department, European Investment Bank, the green bonds’ principles are embedded into four components.

But today the Green Bonds market is at a critical stage in its development. Too unregulated, sometimes opaque, often subject to market imperfections, green bonds represent no more than 5% of bond issues. Some of these challenges are related to ensuring that the use of proceeds from green bonds is strictly guided by sustainability principles to guard against “green washing”. In 2019 emerging market green bond issuance rose from 21% to $52bn with China the largest issuer (Amundi-IFC, 2020). But despite this significant growth, those countries are facing some challenges such as political instabilities, the quality and availability of information and green label assets.

### The current state of low-and middle-income countries’ (LMIC’s) green bonds markets

Markets in low- and middle-income countries are the most exposed regions to climate change risks, but they face an unprecedented challenge to decarbonize their economies while maintaining a sustainable economic development trajectory. The first green bond issuance from LMIC’s took place in 2012 in South Africa, but global growth in these types of bonds is driven by China, with the East Asia and Pacific region accounting for 81% of the market. In 2014, they amounted from $4.5 billion; a 10-fold increase in one year ($42 billion in 2015) to $81 billion in 2016. While green bonds accounted for only 1% of bond issues at the beginning of 2017, growing investor interest and the entry of large bond issuers from the states into this market (previously held by large companies only) should be a game changer (Filkova, 2018).

**Fig. 2:**
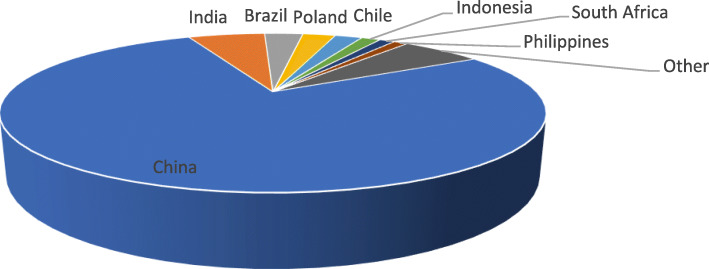
Cumulative LMIC’s Green Bond Issuance from 2012 to 2019 (%)*.* Source: International Financial Corporation, Global Macro & Market Research, Bloomberg, Environmental Finance, Climate Bonds Initiative (2019) https://www.climatebonds.net/files/reports/2019_annual_highlights-final.pdf

Figure 2 shows the share of dominant emerging markets climate-aligned bond issuers from 2012 to 2019. It suggests that China and India dominate the market, and have strengthened growth in this market in 2017. In the first quarter of 2017, the global contribution of emerging markets was 15%, whereas in the first quarter of 2018, this contribution doubled and was 32 of global first-quarter issuance (Filkova, 2018).

### Barriers to the green bond market in LMIC’s

The limits and inconsistency of green bond certification schemes are recognised by all, both by states and international organisations and by market participants. Today, both green bond issuers and investors face the challenge of overcoming the current market turbulence. The global green bond market, which has now exceeded $700 billion in outstanding issues, is booming rapidly. This market is an essential source of financing for projects with positive environmental effects, both in developed and low countries. The main obstacle faced by portfolio managers investing in LMIC’s markets is that ESG standards can be very different from those in Western countries.

However, key challenges are being met to further help the expansion of these green bond markets:

- *Underdeveloped capital markets with insufficient liquidity and high transaction costs*: Promoting green products and greening financial markets are not without challenges for the stock market. These guidelines address a number of specific challenges, obstacles and barriers, including: insufficient supply to meet investor demand; lack of liquidity in green products and taxonomy. By facilitating the issuance, identification and investment of sustainable financial securities, stock exchanges can catalyse the transition of their financial centres while ensuring the sustainability of their activities. In addition, many studies show that investor sentiment may drive decisions (De Long et al., 1990; Cen & Liyan-Yang, 2013) for instance, weather conditions (Hirshleifer & Shumway, 2003; Saunders, 1993).
- *The quality and availability of information to identify, measure and track green investments*: Investors often also seek information about the overall ESG performance of green bond issuers. However, the lack of a generally accepted taxonomy makes it almost impossible to investors and stakeholders to receive complete and reliable information (Filkova & Almeida, 2019).
- *Institutional barriers*: the smallest size and insufficient technical capacity of the financial institutions are some important barriers (Banga, 2019). Indeed, a minimum size of green bond should be reached to ensure liquidity support and tender bonds (Banga, 2019). In some low-income countries profit seeking lead banks to face difficulties in implementing the basics of sustainable finance. There is an extreme need to enhance coöperation within financial institutions so as to develop a sustainable finance roadmap.

### ESG management strengthens its position: what is the contribution of ESG in the bond universe of low- and middle-income countries?

**Fig. 3:**
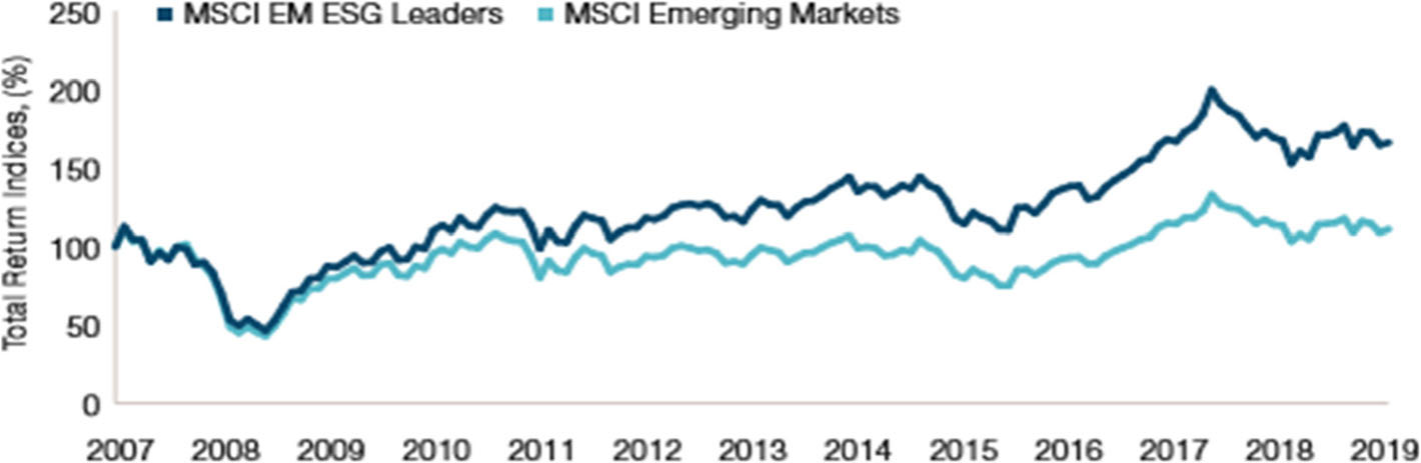
Cumulative Gross Index Return (September 2007 to September 2019). Source: MSCI, data analysis (Rowe Price, 2019)

The 1980s and 1990s were characterized by the creation of several Socially Responsible Investing funds and the launch of the first responsible index with the creation of the Domini Social Index, composed of 400 large US capitalizations selected according to social and environmental criteria. Beginning in the late 1990s, socially responsible investors began to include risk and return considerations in their investment choices through the use of techniques that maximize financial return. This growing interest in the performance of responsible investors led to the UNEP (United Nations Environment Programme) financial initiative in 2003, where a working group on environmental, social and governance (ESG) issues related to financial assets. Environmental, social and governance (ESG) factors, which have long been taken into account in developed economies, are rapidly becoming priority issues in LMIC’s markets. Not surprisingly, over the past decade the MSCI Emerging Markets, which tracks companies that outperform ESG on their peers, outperformed with an annualized gross return of 6.98% (compared to 3.73%) (Rowe Price, 2019) (Fig. 3).

Since the beginning of the coronavirus crisis, some stocks have been able to cushion their fall in the stock market. With the coronavirus pandemic affecting every facet of life, emerging market economies have far less room for fiscal and monetary manoeuvring (Amundi-IFC, 2020).

### Policy recommendations for ESG management in LMIC’s

For LMIC’s green bonds, the limited number of green bonds issuance and the relative lack of proxies, make it relatively difficult to measure the performance of the market (Amundi-IFC, 2020).

### What standards for responsible investing?

To scale up with this issues, some market analysts are suggesting internationally recognized standards. Lack of standardization is a real headache for ESG investors. Indeed, standardization can result in robust frameworks for monitoring, reporting and assurance of the green bond proceeds that will enable new issuers to be attracted by the market opportunities. Unfortunately, a proliferation of standards could “severely slow down” the development of the green bond market. Because today, the lack of clear criteria of definition discourages some issuers from launching for fear of accusation of green washing. On the other hand, the development of overly restrictive standards could have the same deterrent effects. Green bonds are issued within a specific framework, usually the Green Bond Principles (GBP) of International Market Capital Association (2018). Other repositories are sometimes used, but they are generally established by supranational or state agencies. Most self-proclaimed “green” bonds are then given a second opinion by qualified agencies and auditors.

### Taxonomies

As taxonomies are being developed, one issue that’s arising is finding the right balance between. Taxonomies provide all market participants and consumers with a common understanding of qualifying activities, protects against green washing and provides the basis for further policy actions, including standards, labels, incentives and potential changes to prudential rules. Countries will need to determine what works best for their circumstances and how much to develop their own taxonomy or draw on others.

### Building consensus

In December 2019, the Climate Bond Initiative published the third version of its international standard to ensure compatibility with the new European standard for green bonds and the latest version of the Green Bond Principles by strengthening definitions of green bonds and information requirements. The Climate Bond Initiative’s consensus-based standardization and strict taxonomy help pave the way for passive investments and the risk of greenwashing, in other words investments that look greener than they actually are.

### Lower costs and transparency

Improved ESG data collection, processing and standardization allows index providers to codify ESG targets in benchmarks with high accuracy, rigor and transparency. Indeed, managers of actively managed green bond funds charge higher fees to cover research costs and analysts’ salaries. On the other hand, the Exchange-Traded Funds rules-based approach that replicates indices would reduce costs for investors. All investors want to know where their money is going, especially when it comes to ESG investments such as green bonds. In the seeking of transparency, the appointment of mandatory or voluntary external reviewers (to certify alignment with Green Bond Principles, Green Loans Principles, and Climate Bond Standards) increased investors’ confidence.

### Gradually implement sustainable finance and market globalization

Market access is limited in developed countries; indeed, on one hand, to catch up with those countries, the establishment and development of an online learning platform for awareness and training would be an important asset (It could also involve re-rewards for banks, especially local ones, and businesses to encourage strong performance). On the other hand, the improvement of a local market access for an emerging class of global green investors is also a great asset (OECD, 2017).

In order to facilitate the understanding and participation of investors in the African green market, the authorities should first and foremost be receptive to innovation. Second, it is a gradual approach that should include a collaborative multi-party working group to develop guidelines, consultations with all market participants and consumers to develop a national plan, prepare a roadmap, set up a regulatory framework and launch the sector. This involves building an ecosystem for green capital markets, ensuring flexibility taking into account both financial aspects, and economic and environmental contexts. From 2007 to 2016, only 6.5% of global green issuance flowed within low-income countries (Banga, 2019).

### Tax incentives to stimulate market growth

Investors should check the so-called “greenwashing” (directing its actions towards green positioning), the impact on investment, pricing, risk/return trade-offs, and investment comparisons. In the same vein, it is a question of securing a pipeline of market-ready and investment-ready projects for these types of bonds. This requires good Interaction between public officials/private actors to increase climate finance for nationally determined contributions.

### Implications

#### Academic implications

The goal of this paper was to explore emerging research trends of the impact of green bonds in low-and-middle income countries using a systematic approach. This article contributes to research on sustainable finance by elucidating the theoretical evolution of green bonds issuance research and its linkages with multiple economic, social, and governance factors. An understanding of the contributions of the most productive scholars and their research helps to build our work by choosing and following a line of inquiry. This study outlines several research propositions that can serve as a foundation for future research in the area of green bonds issuance.

#### Managerial implications

This study could benefit managers interested in adopting a proactive approach to understand which changes in strategies, services, and products are required to meet unprecedented demands and develop sustainable business practices. The propositions discussed above suggest that, credible standards should be widely applied to develop the market of green bonds in low-and middle-income countries, projects with long-term investments horizons, secure income streams and large capital costs.

## Conclusion

Green bonds are an effective financing mechanism that benefits both issuers and investors, and can help mobilize private capital available in developed countries. Investors are increasingly taking environmental, social and governance factors into account along traditional financial risks. While investments have already been made, significant problems have arisen due to the lack of tools to assess natural resources and in the absence of a strong and universal standard capable of encouraging financial institutions, if not forcing them, to reduce their exposure to the risks associated with climate change. So far, in case of greenwashing, the lack of standardization of emission and monitoring methods are leading many investors to hesitate on the use of ESG criteria for low-and middle-income markets, fearing that this will limit potential opportunities or returns.

This study is trying to gain perceptive insights into the area of sustainable finance research using systematic analysis, and used research scholar databases and institutional reports to collect data. The results suggest that on the contrary to developed countries, the institutional, financial, and political barriers are keeping the green market in low-and-middle income countries in an embryonic state. The results also suggest that measures should be taken to greening the bonds. In their process of greening the financial sector, governments should focus on pension funds and insurance companies; they should increase the level of private sector investment by applying tax liabilities, for example. On the other hand, central banks should harmonize standards to build investor confidence and ease decision-making. This will require the construction of a normative system that is readable and accepted by all. In order to increase the credibility of green bonds in low-and middle-income economies, standardization of reporting is needed in sectors where there is no certification system yet; furthermore, the practice of issuers making hypothetical self-proclaimed statements about green profits (with or without external advice/opinion) must evolve into a model that provides for certification of real profits according to effective and credible standards widely recognized by an accredited third party.

## Data Availability

The datasets analysed during the current study are available in - https://www.ifc.org/wps/wcm/connect/a64560ef-b074-4a53-8173-f678ccb4f9cd/202005-EM-Green-Bonds-Report-2019.pdf?MOD=AJPERES&CVID=n7Gtahg - https://www.troweprice.com/content/dam/ide/articles/pdfs/2019/q4/in-emerging-markets-does-it-pay-to-worry-about-esg-factors.pdf

## Abbreviations

EIB: European Investment Bank
ESG: Environmental, Social and Governance
GBP: Green Bonds Principles
IBRD: International Bank for Reconstruction and Development
LMIC: Low- and Middle-Income Country
MSCI: Morgan Stanley Capital International index
SRI: Socially Responsible Investing
UNEP: United Nations Environment Programme
